# Modeling Music Emotion Judgments Using Machine Learning Methods

**DOI:** 10.3389/fpsyg.2017.02239

**Published:** 2018-01-05

**Authors:** Naresh N. Vempala, Frank A. Russo

**Affiliations:** ^1^SMART Lab, Department of Psychology, Ryerson University, Toronto, ON, Canada; ^2^Toronto Rehabilitation Institute, Toronto, ON, Canada

**Keywords:** music cognition, music emotion, physiological responses, computational modeling, neural networks, machine learning, random forests

## Abstract

Emotion judgments and five channels of physiological data were obtained from 60 participants listening to 60 music excerpts. Various machine learning (ML) methods were used to model the emotion judgments inclusive of neural networks, linear regression, and random forests. Input for models of perceived emotion consisted of audio features extracted from the music recordings. Input for models of felt emotion consisted of physiological features extracted from the physiological recordings. Models were trained and interpreted with consideration of the classic debate in music emotion between cognitivists and emotivists. Our models supported a hybrid position wherein emotion judgments were influenced by a combination of perceived and felt emotions. In comparing the different ML approaches that were used for modeling, we conclude that neural networks were optimal, yielding models that were flexible as well as interpretable. Inspection of a committee machine, encompassing an ensemble of networks, revealed that arousal judgments were predominantly influenced by felt emotion, whereas valence judgments were predominantly influenced by perceived emotion.

## Introduction

The classic philosophical debate on music emotion pits a “cognitivist” view of music emotion against an “emotivist” view (see e.g., Kivy, 1989). The cognitivist view recognizes music as expressing emotion without inducing it in the listener (Konečni, 2008). The emotivist view supposes that music achieves its emotional ends by inducing genuine emotion in the listener. That is to say that the listener not only perceives but also feels the emotion expressed by the music. These feelings may give rise to or be the consequence of physiological responses. Meyer (1956) concedes that while music may on occasion induce a genuine emotional response in the listener, the accompanying physiological responses are likely too undifferentiated to be meaningful.

The debate is far from reconciled, and has been further complicated by the observation that emotion that is perceived in music can in some instances be distinct from emotion that is felt [Gabrielsson, 2002; see Schubert (2014) for a review]. Moreover, Juslin and Västfjäll (2008) argue convincingly that there are likely multiple mechanisms that give rise to felt emotion, ranging from brainstem reflexes to evaluative conditioning. Nonetheless, numerous studies have documented interpretable physiological responses elicited during music listening (Krumhansl, 1997; Nyklicek et al., 1997; Rainville et al., 2006; Lundqvist et al., 2009). Scherer and Zentner (2001) have characterized the cognitivist and emotivist views as complementary, arguing that a fulsome view of music emotion needs to consider both perspectives and the factors that give rise to their dominance.

To the best of our knowledge, the emotivist–cognitivist debate has not been considered from a computational perspective. In the current study, we obtained judgments about emotion conveyed by the music as well as physiological responses. To minimize biasing judgments in favor of one view of music emotion, we told participants that we were interested in judgments of emotion for each excerpt without being explicit regarding “perceived” or “felt” emotion. Judgments were made using a two-dimensional model of emotion encompassing valence and arousal (VA; Russell, 1980). Valence was defined as the hedonic dimension of emotion, ranging from pleasant to unpleasant. Arousal was defined as the mobilization of energy, ranging from calm to excited. In contrast with the discrete view of emotions that argues independent processes for distinct emotions (e.g., Ekman, 1992, 1999), the dimensional approach proposes that all affective states may be characterized on the basis of underlying dimensions of emotion. This approach is in widespread use in music cognition research (e.g., Schubert, 1999; Gomez and Danuser, 2004; Witvliet and Vrana, 2007), and has been found to be particularly effective in characterizing emotionally ambiguous stimuli (Eerola and Vuoskoski, 2011).

**FIGURE 1:**
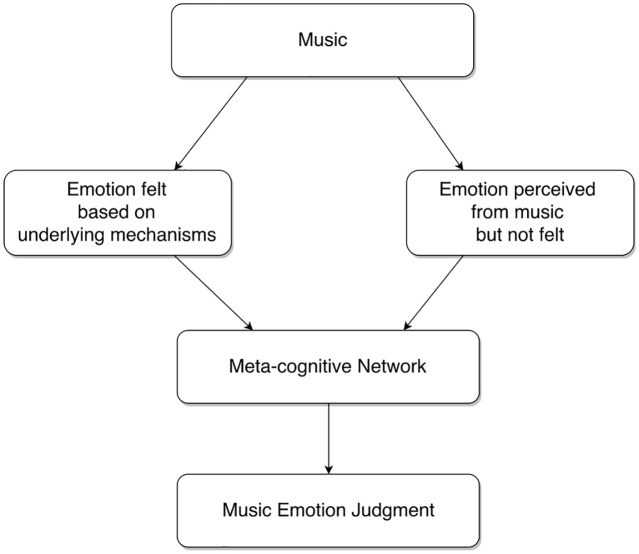
A meta-cognitive network of emotion judgment combining perceived and felt emotion.

We assumed that if the cognitivist position were true, we should be able to model emotion judgments on the basis of deep and surface-level features obtained from the music. Likewise, we assumed that if the emotivist positions were true, we should be able to model emotion judgments on the basis of physiological responses. Another possibility that we considered is that emotion judgments are the result of a meta-level cognitive decision-making process that combines output from a perception module and a feeling module (**Figure 1**). In this scenario, the perception module would take its input from features drawn from the music and the feeling module would take its input from features drawn from physiology. While we acknowledge that this account of emotion judgments is skeletal and reliant on some crude assumptions, it provides a framework to guide our modeling exercise.

We had two main objectives in this study. The first was to develop computational models of emotion judgments. We begin by modeling cognitivist and emotivist positions separately using multilayer perceptrons. We then extend these models to reflect a hybrid position in which both expert networks are considered. We refer to this hybrid, meta-level cognitive framework, as a committee machine^1^. Previous studies have modeled emotion recognition (a) exclusively using audio features [see Kim et al. (2010), for an extensive review; Coutinho and Cangelosi, 2009], (b) exclusively using physiological features (Kim and André, 2008), and (c) using a combination of audio and physiological features in a common network (Coutinho and Cangelosi, 2010). However, none of these studies have modeled emotion recognition as a combination of felt and perceived emotion using a meta-level framework.

Our second objective in this study was more methodological in nature. With the current advent of machine learning (ML), availability and accessibility of ML toolkits, application of ML methods has become more viable for researchers interested in music cognition. While this accessibility to ML methods has opened up new avenues for research, the justification for using specific ML methods is often unclear. In this study, we compared the success of our committee machine with two other ML approaches with the intent of highlighting the relative merits of the different approaches.

## Materials and Methods

### Participants

Our experiment was designed such that it required obtaining emotion judgments and physiological response data from 60 participants. On the basis of previous physiological studies involving testing sessions lasting more than 1 h we were expecting several sources of data loss (e.g., electrodes recording facial muscle activity losing contact due to perspiration). Therefore, we recruited more than the necessary number of participants on an ongoing basis, 110 in total, through our departmental participant pool, until we obtained a complete data set from 60 participants. On average, the final 60 participants (40 females, 15 males, 5 undeclared) were 22.9 years of age (*SD* = 7.2) with 4.0 years of music training (*SD* = 3.9).

### Stimuli and Apparatus

Our stimuli consisted of 60 excerpts of high-quality MIDI music drawn from across four genres – Blues, Metal, Pop, and R&B (15 excerpts per genre). Each excerpt spanned approximately 32 bars in duration. We chose to use MIDI music because of the broad range of meta-level information that may be precisely extracted, consisting of both musical features (e.g., pitch and tempo) and event-related features (e.g., velocity and event onset times), which we plan to use in a separate project.

All 60 excerpts, listed in Appendix 1, were selected such that audio renderings of these MIDI files were representative of their respective genres, and were reasonably consistent with the original versions released commercially. We used MIRtoolbox (Lartillot and Toiviainen, 2007; Lartillot et al., 2008) to extract 12 low-level acoustic to mid-level musical features. These features captured information corresponding to rhythm, timbre, dynamics, pitch, and tonality, and were used in several previous studies on music and emotion (MacDorman et al., 2007; Mion and de Poli, 2008; Laurier et al., 2009; Eerola and Vuoskoski, 2011). The 12 features – *rms*, *lowenergy*, *eventdensity*, *tempo*, *pulseclarity*, *centroid*, *spread*, *rolloff*, *brightness*, *irregularity*, *inharmonicity*, and *mode –* were obtained for each bar of each excerpt (technical descriptions are available in Lartillot, 2014).

Participants used their dominant hand for providing continuous emotion ratings, while their non-dominant hand was connected to the Biopac MP150 data acquisition system for measurement of physiological responses^2^. The five channels of physiological data included heart rate (HR), respiration rate (Resp), skin conductance level (SCL), and facial muscle activity from zygomaticus major (Zyg) and corrugator supercilii (Corr). HR was collected by attaching a photoplethysmogram transducer, using a Velcro strap, to the distal phalange of the middle finger of the non-dominant hand. The transducer was connected to a PPG100C amplifier which measured capillary expansion through an infrared sensor. Resp was measured using a TSD201 respiration belt tightened around the abdomen and attached to an RSP100C amplifier that recorded changes in abdominal circumference. SCL was measured by attaching two TSD203 Ag–AgCl electrodes to the distal phalanges of the index and ring fingers of the non-dominant hand using Velcro straps, connected to a GSR100C amplifier. Facial muscle activity was measured by placing two electrodes over Zyg and two electrodes over corrugator supercilii muscle regions, separated by 25 mm and attached to an EMG100C amplifier.

Physiological data were subjected to feature analysis in order to extract features that have previously been associated with the VA dimensions of emotion. Physiological correlates of valence include Zyg and Corr activity (e.g., Witvliet and Vrana, 2007; Lundqvist et al., 2009; Russo and Liskovoi, 2014). Physiological correlates of arousal include autonomic measures such as HR, respiration, and galvanic skin response (e.g., Iwanaga et al., 1996; Krumhansl, 1997; Baumgartner et al., 2005; Etzel et al., 2006; Sandstrom and Russo, 2010; Russo and Liskovoi, 2014).

### Experimental Design and Data Collection

Our experiment was designed such that (a) each participant listened to 12 of the 60 excerpts (i.e., three from each of four genres) and (b) each excerpt was heard by 12 unique participants. Participants received a listening order that was independently randomized to minimize the influence of presentation order. Each excerpt was preceded by 30 s of white noise and followed by 50 s of silence. The root-mean-square (RMS) level of white noise was equalized with the mean RMS level across all 60 excerpts. White noise was used as our baseline for physiological measurements on the basis of previous studies suggesting the appropriate use of RMS-matched white noise as an emotionally neutral baseline for isolating the effects of emotion on physiology (Nyklicek et al., 1997; Sokhadze, 2007; Sandstrom and Russo, 2010).

Each participant heard a stimulus file with 12 excerpts in randomized order, white noise, and silence in the following sequence:

WN→Ex→S→WN→Ex→S...

Here, WN indicates white noise, Ex indicates excerpt, and S indicates silence. During the silence phase, participants provided familiarity and preference ratings on the excerpt they heard last. All excerpts were presented at approximately 75 dB SPL over Sennheiser HD 580 Precision Headphones. We used the EMuJoy Software (Nagel et al., 2007) for continuous data collection of emotion ratings on the two-dimensional axes of VA (Russell, 1980).

The experimenter provided participants with a description of the two-dimensional model prior to data collection. It was explained that the *x*-axis conveyed emotion ranging from negative to positive (i.e., valence) and the *y*-axis conveyed emotion ranging from calm to excited (i.e., arousal). Participants were asked to continuously rate each excerpt on a two-dimensional grid while listening. Before commencing, listeners familiarized themselves with the EMuJoy interface while listening to two test excerpts that were not included in the formal experiment. After completion of data collection from all 60 participants, mean VA ratings were computed for each participant, for 32 bar-length segments and for the entire excerpt (i.e., the data were averaged per track for each participant). These values were then averaged across the 12 participants to obtain a mean emotion rating profile for that excerpt. This procedure was repeated for all 60 excerpts.

### Data Preparation

Similar to emotion ratings, audio features were extracted for each bar of the excerpt and then aggregated.

Filtering and baseline subtraction for physiological data were performed using FeatureFinder (Andrews et al., 2014), a free Matlab toolbox for physiological signal analysis. The following high-pass (HP) and/or low-pass (LP) filters were applied to raw physiological data: HR (LP = 4 Hz; HP = 0.5 Hz), Resp (LP = 1 Hz; HP = 0.05 Hz), GSR (LP = 10 Hz; HP = 0.5 Hz), Zyg, and Corr (LP = 500 Hz; HP = 5 Hz). Features were obtained for each excerpt and baseline corrected by subtracting the equivalent feature obtained in the final 20 s of 30 s white noise that preceded the excerpt. Similar to audio features and their corresponding emotion ratings, physiological features were computed for each bar of the excerpt and then averaged for its entire duration.

### Machine Learning Models

**FIGURE 2:**
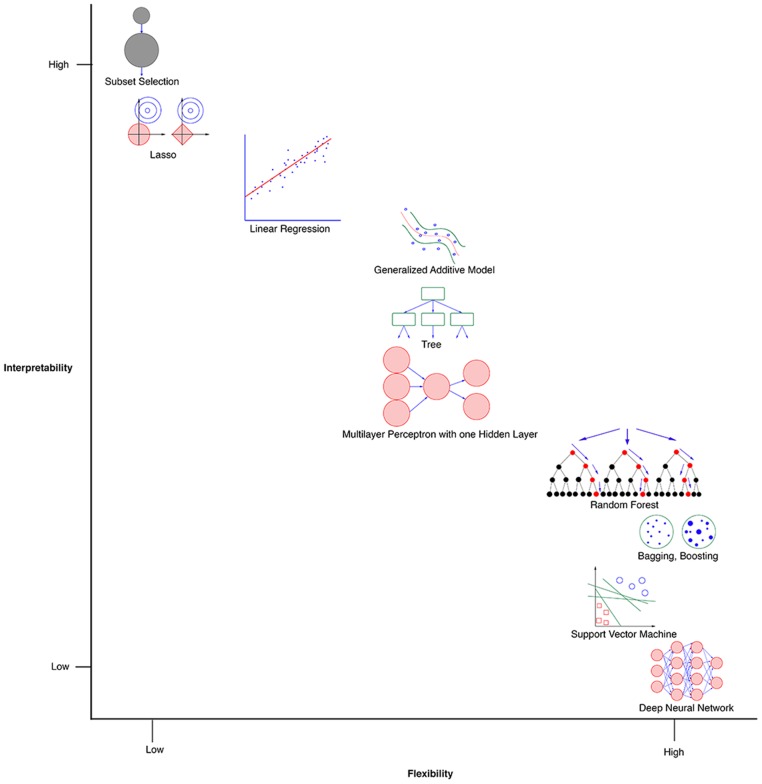
A conceptual visualization of ML methods with respect to interpretability and flexibility, adapted from James et al. (2013).

There exists a multitude of ML methods for both classification and regression. **Figure 2** provides a conceptual visualization that plots flexibility of methods against interpretability of methods. Since our problem involves predicting emotion ratings as opposed to identifying emotion classes, it is a regression problem. There is no single perfectly suited method for a regression problem. In general, models that are developed with methods that are flexible tend to be powerful in terms of fitting the training data (Hastie et al., 2009; James et al., 2013). However, the ability to interpret the salience of features tends to be better in models that have been developed using methods with less flexibility.

Another related issue is that while flexible models can outperform simpler models as regards to reducing training error, they tend to overfit the regression function to the training set. Hence, the performance of models on a given test set can vary dramatically, making their predictions less generalizable. There are two typical ways of addressing this generalizability problem. Option 1 involves starting with methods with low flexibility and then moving toward methods with more flexibility until arriving at a model with good performance and generalizability. Option 2 involves starting with a flexible method that improves the likelihood of arriving at a model with good performance, and then moving toward a simpler method that performs relatively well (Kuhn and Johnson, 2013). We chose to adopt a hybrid approach, starting with a method that typically yields intermediate flexibility (i.e., artificial neural networks), and then progressing to methods with lower or higher flexibility – linear regression and random forests (RFs), respectively.

#### Feature Reduction

**FIGURE 3:**
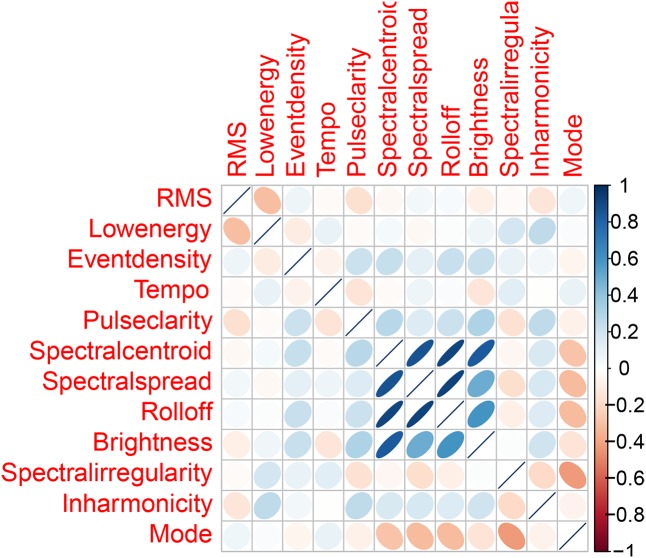
Correlations of the 12 audio features.

When dealing with a high-dimensional dataset, feature reduction by PCA or other means is typically an important step, reducing the storage and computational space while increasing interpretability. In our case, since we were dealing with only 12 audio features, our intention was on the removal of confounding variables. These 12 features serve as independent variables used by our models for predicting the dependent variable – valence or arousal. Although a feature may be strongly correlated with the dependent variable when assessed in isolation, its correlation with the dependent variable may be suppressed when assessed in a model involving numerous features that share common variance. Hence, we computed a correlation matrix of all 12 features. We used a threshold of *r* = |0.8| to remove features that were strongly correlated with each other. Among the four features – *spectral centroid*, *spectral spread*, *rolloff*, and *brightness*, our results (**Figure 3**) showed that *spectral centroid* was strongly correlated with all three features – *spectral spread*, *rolloff*, and *brightness* (*r* > |0.8|, *p* < 0.001) whereas *spectral spread* and *rolloff* were correlated only with two of the remaining three features. *Brightness* was strongly correlated only with spectral centroid. As a result, we chose to remove *spectral centroid* and *rolloff* from our set of features. We also computed a correlation matrix of the five physiological features for all 60 excerpts, with the same threshold of *r* = |0.8| for feature removal. None of the features were strongly correlated with each other. Hence, all five features were retained in our models.

#### Initial Analyses

As a first step in our exploration of the data, we checked to see how well the independent variables accounted for the dependent variables, by examining correlations between the features and the mean VA ratings for the 60 excerpts. We examined correlations for the audio features and physiological features separately since they were being used for separate prediction models.

We observed positive correlations with arousal ratings for *eventdensity*, *r*(58) = 0.48, *p* < 0.005 and brightness, *r*(58) = 0.27, *p* < 0.05. We observed negative correlations with valence ratings for *eventdensity* (*r*(58) = -0.33, *p* < 0.05), *spectral centroid* (*r*(58) = -0.3, *p* < 0.05), and *brightness* (*r*(58) = -0.34, *p* < 0.05). Among the five physiological features, there were no significant correlations with arousal ratings but several with valence. In particular, we observed a negative correlation with valence ratings for Corr, *r*(58) = -0.26, *p* < 0.05, and a positive correlation with valence ratings for Resp, although the latter only reached marginal significance, *r*(58) = -0.24, *p* = 0.06.

#### Artificial Neural Networks

Our objective in modeling was not to merely provide a prediction method for emotion judgments, but to also provide a theoretical explanation for music emotion judgments. Multilayer perceptrons (i.e., a type of artificial neural network) (Rumelhart et al., 1986; Haykin, 2008) have been known to serve as useful connectionist models for exploring theories in cognitive science (see McClelland and Rumelhart, 1989; Vempala, 2014). Our previous work (Vempala and Russo, 2012, 2013; Russo et al., 2013) has shown that multilayer perceptrons with a single hidden layer can lead to nonlinear regression functions for emotion prediction with good explanatory power. Importantly, these models also lend themselves to interpretation.

We implemented three different types of artificial neural network ensembles for predicting emotion judgments of listeners – one that used only audio features from music to model emotion perceived by a listener (perception model), another that used only physiological responses as features to model emotion felt by a listener (feeling model), and a hybrid ensemble that combined outputs from both these network ensembles (hybrid model), henceforth referred to as a committee machine. All the networks were implemented in Matlab. For all three models (i.e., perception model, feeling model, and hybrid model), the dependent variables were the same – VA. The independent variables were audio features for the perception model, and physiological features for the feeling model. Since the hybrid model was a meta-level network that combined outputs from both these models, its independent variables were both audio and physiological features.

We built two networks with audio features as input – one for predicting valence and one for predicting arousal. Each network was a supervised, feedforward network that consisted of 10 input units (i.e., one unit for each feature), one hidden layer, and one output unit for either valence or arousal. One important consideration in the use of neural networks is the propensity to overfit to training data, leading to underperformance when exposed to new data. To make our neural networks more robust, we adopted the following training and testing procedure.

##### Dataset preparation for training and testing

For testing a neural network’s performance, the dataset is usually split into a training set consisting of approximately 70–90% of the data, and a test set consisting of 10–30% of the data, respectively. Some decrease in the network’s performance is expected from the training set to the test set. Poor performance on the test set indicates that the network has either not fully converged while training (i.e., has been under-trained) or has been over-trained. Hence, the network is retrained accordingly. While this is a widely accepted method for validating performance, problems tend to arise because of idiosyncrasies associated with partitioning. In general, some partitions will lead to overfitting while other partitions will lead to underfitting.

To mitigate problems associated with partitioning the dataset, we used *k*-fold cross-validation. Here, the dataset is split into *k* equal-sized partitions called folds. *k* is typically 5 or 10. This allows us to use each of the *k*-folds as a test set with the remaining *k*-1-folds as the training set. The procedure is repeated *k* times. Performance results on all *k*-folds are then averaged. We used fivefold cross-validation, which enabled us to come up with five different trained networks. We separated our dataset of 60 excerpts such that 44 were used for training the models and the remaining 16 were used for testing the models. Forty of the 44 excerpts were partitioned into fivefolds for cross-validation. So, each fold consisted of eight excerpts with two excerpts from each of the four genres. Each of the five networks was trained on 36 excerpts – 32 from the remaining fourfolds along with the additional four excerpts that were not used for cross-validation.

##### Network architecture

For methodological reasons, we used separate networks for predicting VA. This architectural decision enabled us to train networks individually without letting convergence for one dependent variable affect the other. It also allowed us to examine feature salience separately for VA.

**FIGURE 4:**
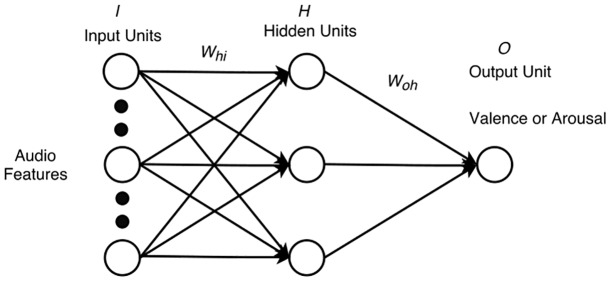
Perception network with 10 features, 3 hidden units, and 1 output.

**FIGURE 5:**
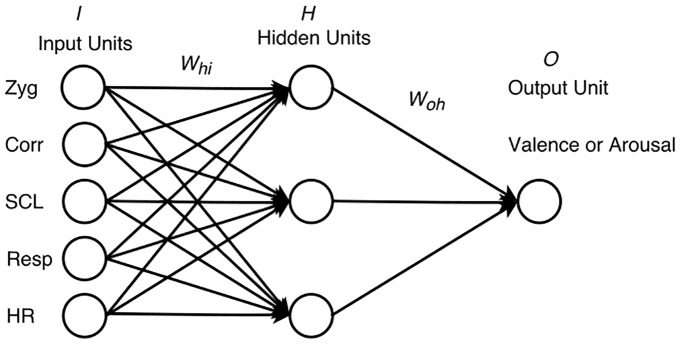
Feeling network with five features, three hidden units, and one output.

The networks had to predict VA ratings based on 10 audio features and/or five physiological features (**Figures 4**, **5**). As such, the training set for each of the networks predicting valence consisted of 36 input vectors and 36 corresponding output values for valence, representing the 36 excerpts. Likewise, the training set for each of the networks predicting arousal consisted of 36 input vectors and 36 corresponding outputs for arousal. For the perception networks, each input vector had 10 values, one for each feature. For the feeling networks, each input vector had five values, one for each physiological feature, collapsed across participants. The corresponding outputs with VA values were again collapsed across participants. To maximize network learning (within and across channels), all of the audio and physiological inputs were scaled to a value between 0 and 1 (Bishop, 1995) for each feature. VA values for all excerpts were obtained on a scale ranging from -1 to 1. To make these values compatible across the networks, we scaled them to a range between 0 and 1. We chose to reduce the number of hidden units to a number that offered us a flexible non-linear solution while minimizing the likelihood of overfitting. To do so, we used an iterative process of trial and error where we started with the number of hidden units equal to the number of input units, then reduced this number by one at each step, while checking to see if the network’s performance remained consistent. Following this process, we decided to keep the number of hidden units to 3. Thus, the network architecture consisted of either 10 input units (one for each audio feature) or five input units (one for each physiological feature), a single hidden layer with three units, and one output unit (either for valence or for arousal).

The following procedure was used to train the network:

(1) Connection weights *W*_hi_ (input units to hidden units) and *W*_oh_ (hidden units to output units) were initialized to random numbers between -0.05 and 0.05. Input vectors were fed to the network from the training set in a randomized order. Inputs were multiplied with the connection weights *W*_hi_, and summed at each hidden unit.
(2) Hidden unit values were obtained by passing the summed value at each hidden unit through a sigmoid function. These values were multiplied with the connection weights *W*_oh_, summed at each output unit, and passed through a sigmoid function to arrive at the final output value between 0 and 1.
(3) Squared errors between the network’s output and the mean valence or arousal rating were computed. The backpropagation algorithm using gradient descent was applied and changes in connection weights were stored. At the end of the entire epoch, connection weights were updated with the sum of all stored weight changes.

The perception networks were trained for approximately 2000–3000 epochs by repeating step (2) to reduce the mean-squared error to less than 0.045. The feeling networks took longer to train than the perception networks, and required approximately 15,000–30,000 epochs of training in order to reduce the mean-squared error to less than 0.045. The learning rate parameter was set to 0.1.

After training, each network was tested on its fold and the root mean-squared error (RMSE) was computed. RMSE values for the audio and physiological networks are shown in **Tables 1**, **2**, respectively. The mean and standard errors for perception and feeling networks, for VA, indicate that both types of networks were more-or-less similar in their averaged performance across the fivefolds.

**Table 1:** RMSE values of the five perception networks.

Fold	Valence RMSE	Arousal RMSE
1	0.27	0.18
2	0.21	0.34
3	0.16	0.33
4	0.26	0.14
5	0.16	0.15
Mean	0.21	0.23
SE	0.03	0.05

*SE indicates standard error.*

**Table 2:** RMSE values of the five feeling networks.

Fold	Valence RMSE	Arousal RMSE
1	0.26	0.25
2	0.24	0.33
3	0.19	0.29
4	0.23	0.24
5	0.24	0.35
Mean	0.23	0.29
SE	0.01	0.02

*SE indicates standard error.*

##### Performance of perception and feeling networks

After completing network training, we tested the trained networks on the remaining 16 excerpts. We used all five perception networks together as an ensemble and averaged their outputs to give the final output for each test excerpt, for VA. We used the same procedure to compute outputs from the feeling networks. For valence, RMSE values for the perception network ensemble and the feeling network ensemble were 0.27 and 0.34, respectively, suggesting that the perception networks performed better than the feeling networks in predicting valence. For arousal, RMSE values for the perception network ensemble and the feeling network ensemble were 0.24 and 0.23, respectively, suggesting that both networks performed similarly.

##### Committee machine

**FIGURE 6:**
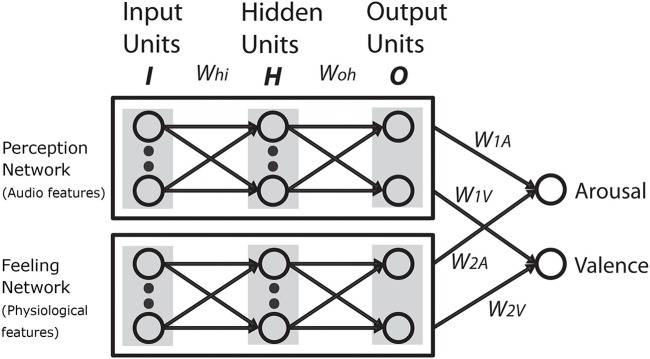
Committee machine – a hybrid network combining results from the perception and feeling network ensembles.

Our next step was to build a model under the assumption that (a) listeners make separate emotion assessments based on what they perceive from the music and what they feel when listening, and (b) their final appraisal of emotion is based on a weighted judgment that takes contributions from both sources into account. This led us to implement our final hybrid model – a committee machine (Haykin, 2008). The committee machine is a meta-level network, as shown in **Figure 6**, which combines outputs from each individual ensemble to arrive at its final output.

First, we implemented a basic committee machine, which merely averaged the outputs from both network ensembles. Specifically, when predicting either the valence or the arousal of an excerpt, outputs from the perception network ensemble and the feeling network ensemble were combined with equal weight contributions of 0.5. RMSE values for the committee machine with ensemble averaged weights (CMEA) were 0.28 for valence and 0.21 for arousal. These results indicate that for valence, the basic committee machine performed about as well as the perception networks and better than the feeling networks. However, for arousal, the basic committee machine performed better than both perception and feeling networks.

Next, we implemented a committee machine that was consistent with our hybrid framework where weights from each of these network ensembles contributed to the final emotion judgment in a way that illustrated meta-level decisions based on emotion conveyed by perception and feeling. To obtain an optimal linear combination of the weights (Hashem, 1997) from each of these individual network ensembles, we performed multiple linear regression such that outputs from these individual ensembles were used as independent variables and mean VA ratings were used as dependent variables. Linear regression was performed on the entire set of 60 excerpts. The models for VA are provided in Equations (1) and (2), respectively.

(1) $$y\text{V}=0.757x_{1\text{V}}+0.164x_{2\text{V}}+0.056$$

(2) $$y\text{A}=0.813x_{1\text{A}}+0.968x_{2\text{A}}-0.396$$

Here, *y*_V_ and *y*_A_ refer to the VA outputs of the committee machine on a scale from 0 to 1. *x*_1V_ and *x*_1A_ refer to the VA outputs from the perception network ensemble on a scale from 0 to 1. Likewise, *x*_2V_ and *x*_2A_ refer to the VA outputs from the feeling network ensemble on a scale from 0 to 1.

Based on Equation (1), for valence, the meta-level network applies a weight of 0.757 to the perception ensemble output, 1.164 to the feeling ensemble output, and has a bias unit of weight 0.056. Likewise, for arousal, based on Equation (2) the meta-level network applies a weight of 0.813 to the perception ensemble output, 0.968 to the feeling network output, and has a bias unit of weight -0.396. To understand the salience of each individual network’s contribution to the overall prediction, we computed their proportion contributions while ignoring the intercepts. For valence, the weight contributions were 82.2% from the perception ensemble and 17.8% from the feeling ensemble. For arousal, the weight contributions were 45.6% from the perception ensemble and 54.4% from the feeling ensemble. As expected, this committee machine (CMLR) performed better than the individual ensembles, and the CMEA, with RMSE values of 0.26 for valence, and 0.2 for arousal.

#### Linear Regression

Although neural networks helped us from the perspective of cognitive modeling, we wanted to ensure from the perspective of ML that neural networks were not too powerful for our needs. Perhaps a simpler and more interpretable approach could predict VA ratings just as well. To mitigate the possibility of overfitting and to allow for a consistent comparison between models obtained from different ML methods, we again used fivefold cross-validation with the same 44 excerpts that were used for our neural networks. We performed stepwise forward regression to examine which of the 10 audio features were strongly correlated with the VA ratings. The stepwise criteria in our regression models included variables which increased probability of *F* by at least 0.05, and excluded variables which decreased probability of *F* by less than 0.1. This led to four derived regression models that predicted valence, and five derived regression models that predicted arousal, using audio features.

For valence, the first model accounted for 17.9% of the variance in ratings, *F*(1,34) = 7.39, *p* < 0.05. The model contained only brightness as its predictor variable (*p* < 0.05). The second model accounted for 18.3% of the variance in ratings, *F*(1,34) = 7.63, *p* < 0.01. Again, the model contained only brightness as its predictor variable (*p* < 0.01). The third model accounted for 25.4% of the variance in ratings, *F*(2,33) = 5.62, *p* < 0.01. The model contained brightness and lowenergy as its predictor variables (*p* < 0.01, *p* < 0.05, respectively). The fourth model accounted for 36.9% of the variance in ratings, *F*(3,32) = 6.24, *p* < 0.01. The model contained brightness, lowenergy, and mode as its predictor variables (*p* < 0.01, *p* < 0.05, and *p* < 0.05, respectively).

For arousal, the first model accounted for 33.4% of the variance in ratings, *F*(1,34) = 17.02, *p* < 0.001. The model contained only eventdensity as its predictor variable (*p* < 0.001). The second model accounted for 43.9% of the variance in ratings, *F*(1,34) = 26.6, *p* < 0.001. The model contained only eventdensity as its predictor variable (*p* < 0.001). The third model accounted for 39.0% of the variance in ratings, *F*(2,33) = 10.6, *p* < 0.001. The model contained eventdensity and mode as its predictor variables (*p* < 0.01 and *p* < 0.05, respectively). The fourth model accounted for 23.6% of the variance in ratings, *F*(1,34) = 10.5, *p* < 0.01. The model contained only eventdensity as its predictor variable (*p* < 0.01). The fifth model accounted for 28.5% of the variance in ratings, *F*(1,34) = 13.6, *p* < 0.01. Again, the model contained only eventdensity as its predictor variable (*p* < 0.01).

We performed stepwise forward regression with the same criteria as before, using the five physiological features as our predictors. This led to three derived regression models that predicted valence. No significant model emerged for arousal.

For valence, the first model accounted for 12.8% of the variance in ratings, *F*(1,34) = 5.01, *p* < 0.05. The model contained only Corr as its predictor variable (*p* < 0.05). The second model accounted for 14.4% of the variance in ratings, *F*(1,34) = 5.72, *p* < 0.05. The model contained only Corr as its predictor variable (*p* < 0.05). The third model accounted for 24.9% of the variance in ratings, *F*(1,34) = 11.28, *p* < 0.01. Again, the model contained only Corr as its predictor variable (*p* < 0.01).

We tested these linear regression models on the 16 excerpts, which the networks had previously not been exposed to. We used all four perception models for valence and all five perception models for arousal as ensembles by averaging their outputs to give the final output for each test excerpt. We used the same procedure for averaging outputs from the three feeling models for valence. For valence, RMSE values for the perception ensemble and the feeling ensemble were 0.25 and 0.66, respectively, clearly showing that the perception ensemble performed much better than the feeling ensemble in predicting valence. For arousal, a comparison between perception and feeling ensembles could not be made since no significant model emerged using physiological features. RMSE for the perception ensemble was 0.23. These results indicate that with audio features, a linear model was sufficient to achieve prediction performance similar to a more flexible model such as a neural network; however, with physiology features, a flexible, nonlinear ML model was necessary to capture the predictive capacity of the independent variables.

#### Random Forests

Our next step was to see if an approach to modeling with greater flexibility than neural networks could lead to better performance. To reiterate, we were interested in whether a different ML model could offer better prediction, ignoring its suitability as a cognitive computational model. We used RFs (Hastie et al., 2009; James et al., 2013) for this purpose, and implemented them using the *caret* (Kuhn et al., 2016) and *mlbench* (Leisch and Dimitriadou, 2010) packages in R. Random forests create an ensemble of decision trees. Features from the available list are randomly selected with replacement to first construct individual decision trees using the training data. After several such decision trees are constructed, whenever a new sample is fed to the random forest, predictions are made by these trees. The mean of all predictions is used as the bagged final prediction of the random forest. So, RFs, by nature, are an ensemble method, and are therefore useful for reducing error due to overfitting. An additional aspect of RFs is that they repeatedly take bootstrapped samples from the training data, with replacement, to construct decision trees. This process also helps in reducing error due to overfitting. As such, splitting the data using *k*-fold cross-validation is considered to be unnecessary.

Again, we trained separate random forest models for VA using audio features and physiology features and tested these trained models on the 16 test excerpts. For valence, RMSE values for the perception model and the feeling model were 0.25 and 0.28, respectively, displaying the same pattern as before, with perception features enabling better performance than feeling features. For arousal, RMSE values for the perception model and the feeling model were 0.2 and 0.26, respectively, suggesting that the perception model had an advantage.

As seen in **Table 3**, the Random Forest models obtained using audio features or physiological features were comparable in performance to the committee machine derived using an ensemble of neural networks.

**Table 3:** Summary of all ML model results.

	Machine learning methods					
	Neural networks		Multiple linear regression		Random forests	
	Valence	Arousal	Valence	Arousal	Valence	Arousal
Audio features (perception models)	Five trained models from fivefold cross-validation (44 excerpts)	Five trained models from fivefold cross-validation (44 excerpts)	Four trained models from fivefold cross-validation (44 excerpts)	Five trained models from fivefold cross-validation (44 excerpts)		
Ensemble performance (16 excerpts) RMSE	0.27	0.24	0.25	0.23	0.25	0.20
Physiology features (feeling models)	Five trained models from fivefold cross-validation (44 excerpts)	Five trained models from fivefold cross-validation (44 excerpts)	Three trained models from fivefold cross-validation (44 excerpts)	No model		
Ensemble performance (16 excerpts) RMSE	0.34	0.23	0.66	No model	0.28	0.26
Committee Machine – CMLR (16 excerpts) RMSE	0.26	0.20				

## Discussion

In this study, we revisited the classic debate on music and emotion involving the cognitivists and the emotivists. We approached the debate from a computational modeling perspective by using neural networks (multilayer perceptrons). We modeled emotion judgments from the cognitivist perspective using deep and surface-level audio features obtained from the music alone. Likewise, we modeled emotion judgments from the emotivists perspective using features that relate to felt emotion (i.e., physiological responses). Both networks performed similarly for arousal. However, for valence, the perception networks (i.e., cognitivist) performed better than the feeling networks (i.e., emotivist).

We also proposed another possibility that emotion judgments can be modeled as a meta-level cognitive decision-making process that combines output from a perception module and a feeling module (**Figure 1**) – a hybrid of the cognitivist and emotivist positions. In this scenario, a perception module takes its input from features drawn from the music, while a feeling module takes its input from features drawn from listener physiology. We modeled this possibility using a committee machine that combined VA contributions from two separate network ensembles – a perception network ensemble and a feeling network ensemble. The committee machine performed better than the individual ensembles.

The committee machine enabled us to understand the contribution of each individual network ensemble. For valence, the weight contributions were 82.2% from the perception ensemble and 17.8% from the feeling ensemble. For arousal, the weight contributions were 45.6% from the perception ensemble and 54.4% from the feeling ensemble. From a theoretical perspective, these findings suggest that felt emotion is more salient in arousal judgments and that perceived emotion is more salient in valence judgments. Given that the feeling ensemble consists of physiological features, and contributed more toward arousal than the perception ensemble, these findings also support the current view in the field about the tight correspondence between physiological features and the arousal dimension of emotion.

We also assessed the validity of our ML method (i.e., neural networks) used for building the committee machine, by comparing it with two other ML methods – multiple linear regression and RFs. To keep comparisons between ML methods consistent, we used the same partitioning of data for training and testing with fivefold cross-validation. This comparison allowed us to ensure that we found the right balance between feature interpretability and model flexibility with neural networks. Multiple linear regression while being less flexible than neural networks as a regression method afforded us the ability to interpret features better. However, this approach revealed its own limitations associated with lack of flexibility. We found that linear methods were not sufficient for deriving a robust, generalizable regression function, using physiological features. When physiological features were used individually as predictors, they were not able to yield a regression model with significant predictors. We refer to these cases as “no model,” indicating that none of the features satisfied the inclusion criteria as predictors in a regression model. However, when the features were used in combination with each other as a nonlinear regression function within neural networks, they performed as well or better than audio features in predicting arousal. We chose RFs as our third method, since they are a highly flexible ML method offering various benefits (i.e., building decision trees through binary recursion, repeated subsampling of features and training data to create variance, and ensemble averaging of trees to avoid overfitting). Despite these advantages, the RF approach did not lead to models with greater explanatory power than that which was obtained using neural networks.

There are several important limitations to this work. First, it is important to acknowledge that we cannot fully isolate features that reflect felt emotion as distinct from those that reflect perceived emotion. In all likelihood, the perception of emotion influences the feeling of emotion, independent of the way in which these two networks eventually combine at the level of cognition. Future work should attempt to reconcile this important detail. As we noted at the outset, the models considered here are skeletal and built upon some rather crude assumptions. Second, we have no way of assessing the quality of the features that we provided to the models. The audio features considered as input in the perception models may or may not have been a subset of the full profile of features that were actually processed by listeners. Similarly, although the physiological features we extracted are clearly associated with felt emotion, they do not likely represent the full profile of neurobiological features underlying felt emotion. Accordingly, the power of all of the networks considered here should be considered as bounded by the decisions that were made regarding inputs. Finally, our modeling attempts were handicapped by the size of our dataset. We noticed correlations between some of the physiological features and arousal in some of the genres considered. However, the size of these correlations was reduced when the entire dataset was modeled. Since each genre was limited to 15 excerpts, models derived at the genre level should be interpreted with caution due to concerns about generalizability.

## Ethics Statement

All participants gave written informed consent in accordance with the Declaration of Helsinki. The protocol was approved by the Ryerson Research Ethics Board (REB# 2012-343).

## Author Contributions

NV was responsible for design, data collection, analysis, modeling, and writing. FR was responsible for design, analysis, and writing.

## Conflict of Interest Statement

The research was co-sponsored by WaveDNA, an industry partner. Although the manuscript presents no opportunity for commercial promotion (there was no use or evaluation of commercial products), it is possible that some version of the computational models described here will be integrated into future releases of WaveDNA’s commercial software. The authors declare that the research was conducted in the absence of any commercial or financial relationships that could be construed as a potential conflict of interest.

## Footnotes

1 A preliminary version of the committee machine described here was reported in Vempala and Russo (2013). Although this prior work was informed by the same theoretical framework, the computational model was based on only 12 excerpts of classical music. Given this small number of excerpts and the lack of genre diversity, the generalizability of the model was extremely limited.

2 The current study utilizes mean responses (emotion judgments and physiological responses); continuous ratings will be modeled in a separate study.

## References

[B1] Andrews A. J. Nespoli G. Russo F. A. (2014). *FeatureFinder (Version 2.5)*. Available at: http://www.featurefinder.ca/

[B2] Baumgartner T. Esslen M. Jäncke L. (2005). From perception to emotion experience: Emotions evoked by pictures and classical music. *Int. J. Psychophysiol.* 60 34–43. 10.1016/j.ijpsycho.2005.04.007 15993964

[B3] Bishop C. M. (1995). *Neural Networks for Pattern Recognition.* New York, NY: Oxford University Press.

[B4] Coutinho E. Cangelosi A. (2009). The use of spatio-temporal connectionist models in psychological studies of musical emotions. *Music Percept.* 29 359–375. 10.1525/mp.2009.27.1.1

[B5] Coutinho E. Cangelosi A. (2010). A neural network model for the prediction of musical emotions. *Adv. Cogn. Syst.* 71 333 10.1049/pbce071e_ch12

[B6] Eerola T. Vuoskoski J. K. (2011). A comparison of the discrete and dimensional models of emotion in music. *Psychol. Music* 39 18–49. 10.1177/0305735610362821 25813790

[B7] Ekman P. (1992). Are there basic emotions. *Psychol. Rev.* 99 550–553. 10.1037/0033-295X.99.3.550 1344638

[B8] Ekman P. (1999). “Basic emotions,” in *Handbook of Cognition and Emotion*, eds Dalgleish T. Power M. J. (New York, NY: John Wiley & Sons), 45–60.

[B9] Etzel J. A. Johnsen E. L. Dickerson J. Tranel D. Adolphs R. (2006). Cardiovascular and respiratory responses during musical mood induction. *Int. J. Psychophysiol.* 61 57–69. 10.1016/j.ijpsycho.2005.10.025 16460823

[B10] Gabrielsson A. (2002). Emotion perceived and emotion felt: same or different? *Music. Sci.* 5 123–147.

[B11] Gomez P. Danuser B. (2004). Affective and physiological responses to environmental noises and music. *Int. J. Psychophysiol.* 53 91–103. 10.1016/j.ijpsycho.2004.02.002 15210287

[B12] Hashem S. (1997). Optimal linear combinations of neural networks. *Neural Netw.* 10 599–614. 10.1016/S0893-6080(96)00098-6 12662858

[B13] Hastie T. Tibshirani R. Friedman J. (2009). *The Elements of Statistical Learning: Data Mining, Inference and Prediction*, 2nd Edn. New York, NY: Springer.

[B14] Haykin S. (2008). *Neural Networks and Learning Machines*, 3rd Edn. Upper Saddle River, NJ: Prentice Hall.

[B15] Iwanaga M. Ikeda M. Iwaki T. (1996). The effects of repetitive exposure to music on subjective and physiological responses. *J. Music Ther.* 33 219–230. 10.1093/jmt/33.3.219 24999623

[B16] James G. Witten D. Hastie T. Tibshirani R. (2013). *An Introduction to Statistical Learning: With Applications in R*. New York, NY: Springer.

[B17] Juslin P. N. Västfjäll D. (2008). Emotional responses to music: the need to consider underlying mechanisms. *Behav. Brain Sci.* 31 559–621. 10.1017/S0140525X08005293 18826699

[B18] Kim J. André E. (2008). Emotion recognition based on physiological changes in music listening. *IEEE Trans. Pattern Anal. Mach. Intell.* 30 2067–2083. 10.1109/TPAMI.2008.26 18988943

[B19] Kim Y. E. Schmidt E. M. Migneco R. Morton B. G. Richardson P. Scott J. (2010). “Music emotion recognition: a state of the art review,” in *Proceedings of the 11th International Society for Music Information Retrieval Conference (ISMIR), Utrecht* 255–266.

[B20] Kivy P. (1989). *Sound Sentiment: An Essay on the Musical Emotions, Including the Complete Text of the Corded Shell*. Philadelphia, PA: Temple University Press.

[B21] Konečni V. J. (2008). Does music induce emotion? A theoretical and methodological analysis. *Psychol. Aesthet. Creat. Arts* 2 115–129. 10.1037/1931-3896.2.2.115

[B22] Krumhansl C. (1997). An exploratory study of musical emotions and psychophysiology. *Can. J. Exp. Psychol.* 51 336–352. 10.1037/1196-1961.51.4.336 9606949

[B23] Kuhn M. Johnson K. (2013). *Applied Predictive Modeling.* New York, NY: Springer.

[B24] Kuhn M. Wing J. Weston S. Williams A. Keefer C. Engelhardt A. (2016). *Caret: Classification and Regression Training, R package version 6.0–64.* Available at: https://github.com/topepo/caret/

[B25] Lartillot O. (2014). *MIRtoolbox 1.6.1 User’s Manual.* Technical report, Aalborg: Aalborg University.

[B26] Lartillot O. Toiviainen P. (2007). “A Matlab toolbox for musical feature extraction from audio,” in *Proceedings of the 10th International Conference on Digital Audio Effects*, Bordeaux, 237–244.

[B27] Lartillot O. Toiviainen P. Eerola T. (2008). “A matlab toolbox for music information retrieval,” in *Data Analysis, Machine Learning and Applications. Studies in Classification, Data Analysis, and Knowledge Organization*, eds Preisach C. Burkhardt H. Schmidt-Thieme L. Decker R. (Berlin: Springer), 261–268.

[B28] Laurier C. Lartillot O. Eerola T. Toiviainen P. (2009). “Exploring relationships between audio features and emotion in music,” in *Proceedings of the 7th Triennial Conference of European Society for the Cognitive Sciences of Music (ESCOM 2009)*, Jyväskylä.

[B29] Leisch F. Dimitriadou E. (2010). *Mlbench: Machine Learning Benchmark.* R package version 2.1–1.

[B30] Lundqvist L. Carlsson F. Hilmersson P. Juslin P. N. (2009). Emotional responses to music: experience, expression, and physiology. *Psychol. Music* 37 61–90. 10.1177/0305735607086048

[B31] MacDorman K. F. Ough S. Ho C. C. (2007). Automatic emotion prediction of song excerpts: index construction, algorithm design, and empirical comparison. *J. New Music Res.* 36 281–299. 10.1080/09298210801927846

[B32] McClelland J. L. Rumelhart D. E. (1989). *Explorations in Parallel Distributed Processing: A Handbook of Models, Programs, and Exercises.* Cambridge, MA: MIT press.

[B33] Meyer L. (1956). *Emotion and Meaning in Music.* Chicago, IL: University of Chicago Press.

[B34] Mion L. de Poli G. (2008). Score-independent audio features for description of music expression. *IEEE Trans. Audio Speech Lang. Process.* 16 458–466. 10.1109/TASL.2007.913743

[B35] Nagel F. Kopiez R. Grewe O. Altenmüller E. (2007). EMuJoy: software for continuous measurement of perceived emotions in music. *Behav. Res. Methods* 39 283–290. 10.3758/BF03193159 17695356

[B36] Nyklicek I. Thayer J. F. Van Doornen L. J. P. (1997). Cardiorespiratory differentiation of musically-induced emotions. *J. Psychophysiol.* 11 304–321.

[B37] Rainville P. Bechara A. Naqvi N. Damasio A. R. (2006). Basic emotions are associated with distinct patterns of cardiorespiratory activity. *Int. J. Psychophysiol.* 61 5–18. 10.1016/j.ijpsycho.2005.10.024 16439033

[B38] Rumelhart D. E. Hinton G. E. Williams R. J. (1986). Learning representations by back-propagating errors. *Nature* 323 533–536. 10.1038/323533a0

[B39] Russell J. A. (1980). A circumplex model of affect. *J. Pers. Soc. Psychol.* 39 1161–1178. 10.1037/h0077714

[B40] Russo F. A. Liskovoi L. (2014). “Physiological responses,” in *Music in the Social and Behavioral Sciences: An Encyclopedia*, ed. Thompson W. F. (London: SAGE Publications), 862–865.

[B41] Russo F. A. Vempala N. N. Sandstrom G. M. (2013). Predicting musically induced emotions from physiological inputs: linear and neural network models. *Front. Psychol.* 4:168. 10.3389/fpsyg.2013.00468 23964250 PMC3737459

[B42] Sandstrom G. M. Russo F. A. (2010). Music hath charms: the effects of valence and arousal on the regulation of stress. *Music Med.* 2 137–143. 10.1177/1943862110371486

[B43] Scherer K. R. Zentner M. R. (2001). “Emotional effects of music: production rules,” in *Series in Affective Science. Music and Emotion: Theory and Research*, eds Juslin P. N. Sloboda J. A. (New York, NY: Oxford University Press).

[B44] Schubert E. (1999). *Measurement and Time-Series Analysis of Emotion in Music.* Ph.D. thesis, University of New South Wales, Sydney, NSW.

[B45] Schubert E. (2014). Emotion felt by the listener and expressed by the music: literature review and theoretical perspectives. *Front. Psychol.* 4:837. 10.3389/fpsyg.2013.00837 24381565 PMC3865445

[B46] Sokhadze T. (2007). Effects of music on the recovery of autonomic and electrocortical activity after stress induced by aversive visual stimuli. *Appl. Psychophysiol. Biofeedback* 32 31–50. 10.1007/s10484-007-9033-y 17333313

[B47] Vempala N. N. (2014). “Neural network models,” in *Music in the Social and Behavioral Sciences: An Encyclopedia*, ed. Thompson W. F. (London: SAGE Publications), 805–807.

[B48] Vempala N. N. Russo F. A. (2012). “Predicting emotion from music audio features using neural networks,” in *Proceedings of the 9th International Symposium on Computer Music Modeling and Retrieval (CMMR)*, London.

[B49] Vempala N. N. Russo F. A. (2013). “Exploring cognitivist and emotivist positions of musical emotion using neural network models,” in *Proceedings of the 12th International Conference on Cognitive Modeling (ICCM)*, Ottawa, ON.

[B50] Witvliet C. V. Vrana S. R. (2007). Play it again Sam: repeated exposure to emotionally evocative music polarises liking and smiling responses, and influences other affective reports, facial EMG, and heart rate. *Cogn. Emot.* 21 3–25. 10.1080/02699930601000672

