# Supplementary material for: Modeling Music Emotion Judgments Using Machine Learning Methods
Source: Front Psychol. 2018 Jan 5;8:2239. doi: 10.3389/fpsyg.2017.02239 (PMC5760560; doi:10.3389/fpsyg.2017.02239)
Supplement: Supplementary file 1 [file Appendix.pdf]

Appendix: List of 60 excerpts and their normalized valence and arousal judgments.

Excerpt	Genre	Title	Artist	Mean Valence	Standard Deviation	Mean Arousal	Standard Deviation
1	Blues	A Good Fool Is Hard To Find	Albert Collins	0.19	0.31	0.33	0.29
2	Blues	Ain't No Tellin'	Mississippi John Hurt	0.33	0.38	0.01	0.22
3	Blues	Born Under A Bad Sign	Albert King	0.12	0.31	0.20	0.30
4	Blues	Get Off My Back Woman	B.B. King	0.14	0.35	0.07	0.45
5	Blues	The Thrill Is Gone	B.B. King	0.27	0.48	-0.16	0.44
6	Blues	To Know You Is To Love You	B.B. King	0.17	0.35	-0.06	0.39
7	Blues	Key To The Highway	Big Bill Broonzy	0.01	0.42	-0.16	0.38
8	Blues	Caught In The Crossfire	Stevie Ray Vaughan	0.05	0.41	-0.11	0.23
9	Blues	I Believe I'll Dust My Broom	Robert Johnson	0.38	0.38	0.24	0.40
10	Blues	Before You Accuse Me	Eric Clapton	0.45	0.32	0.25	0.42
11	Blues	Blues Power	Eric Clapton	0.06	0.48	0.13	0.50
12	Blues	Good Morning Little School Girl	Sonny Boy Williamson	0.10	0.42	-0.11	0.37
13	Blues	Help Me	Sonny Boy Williamson	0.20	0.41	0.17	0.34
14	Blues	Help Me Through The Day	Freddie King	0.35	0.59	-0.19	0.42
15	Blues	Hideaway	Freddie King	0.21	0.44	0.13	0.44
16	Metal	Children Of The Grave	Black Sabbath	-0.28	0.3	0.21	0.41
17	Metal	Heaven And Hell	Black Sabbath	-0.11	0.35	0.22	0.44
18	Metal	Nativity In Black	Black Sabbath	0.22	0.42	0.34	0.3
19	Metal	Paranoid	Black Sabbath	0.15	0.27	0.35	0.48
20	Metal	Killers	Iron Maiden	0.23	0.22	0.52	0.26
21	Metal	Wasted Years	Iron Maiden	-0.09	0.41	0.20	0.56
22	Metal	Metal Meltdown	Judas Priest	-0.15	0.48	0.33	0.52
23	Metal	God of Thunder	Kiss	0.04	0.43	0.36	0.43
24	Metal	The Memory Remains	Metallica	-0.03	0.35	0.15	0.45
25	Metal	Enter Sandman	Metallica	0.10	0.58	0.35	0.50
26	Metal	Dr. Feelgood	Mötley Crew	-0.20	0.50	-0.02	0.46
27	Metal	Crazy Train	Ozzy Osbourne	0.12	0.32	0.39	0.38
28	Metal	Becoming	Pantera	-0.01	0.38	0.39	0.40
29	Metal	Cowboys From Hell	Pantera	0.18	0.40	0.45	0.39
30	Metal	Walk	Pantera	-0.05	0.50	0.34	0.39
31	Pop	A Woman's Worth	Alicia Keys	0.26	0.48	-0.33	0.25
32	Pop	Crazy In Love	Beyoncé	0.44	0.23	0.55	0.32
33	Pop	Halo	Beyoncé	0.39	0.54	0.08	0.31
34	Pop	U + Ur Hand	Pink	0.04	0.59	0.21	0.26
35	Pop	I'm Like A Bird	Nelly Furtado	0.16	0.40	0.04	0.22
36	Pop	Waiting For Tonight	Jennifer Lopez	0.43	0.24	0.27	0.43
37	Pop	California Gurls	Katy Perry	0.31	0.55	0.26	0.44
38	Pop	Thinking Of You	Katy Perry	0.08	0.29	-0.24	0.43
39	Pop	Miss Independent	Kelly Clarkson	0.23	0.29	-0.05	0.28
40	Pop	Born This Way	Lady Gaga	0.29	0.28	0.39	0.20
41	Pop	Just Dance	Lady Gaga	0.33	0.42	0.47	0.49

42	Pop	Better In Time	Leona Lewis	0.21	0.49	-0.07	0.25
43	Pop	Numb	Linkin Park	0.20	0.43	0.27	0.46
44	Pop	Makes Me Wonder	Maroon 5	0.32	0.32	0.33	0.28
45	Pop	Get The Party Started	Pink	0.06	0.53	0.24	0.52
46	R&B/Soul	You Know I'm No Good	Amy Winehouse	0.24	0.38	0.04	0.39
47	R&B/Soul	Rehab	Amy Winehouse	0.33	0.29	0.24	0.28
48	R&B/Soul	Cant Get Enough Of Your Love	Barry White	0.29	0.42	0.21	0.42
49	R&B/Soul	Shining Star	Earth, Wind & Fire	0.49	0.20	0.49	0.13
50	R&B/Soul	Boogie Wonderland	Earth, Wind & Fire	0.10	0.45	0.14	0.36
51	R&B/Soul	Higher Ground	Stevie Wonder	0.30	0.28	0.34	0.27
52	R&B/Soul	How Come You Don't Call Me	Alicia Keys	0.16	0.46	-0.15	0.40
53	R&B/Soul	I Wish It Would Rain	The Temptations	0.37	0.38	-0.16	0.43
54	R&B/Soul	Papa's Got A Brand New Bag	James Brown	0.16	0.43	0.20	0.39
55	R&B/Soul	Just The Two Of Us	Bill Withers	0.40	0.36	0.03	0.52
56	R&B/Soul	Lady Marmalade	Christina Aguilera	0.20	0.42	0.19	0.44
57	R&B/Soul	Lets Stay Together	Al Green	0.23	0.48	0.01	0.43
58	R&B/Soul	I Heard It Through The Grapevine	Marvin Gaye	0.25	0.27	0.11	0.31
59	R&B/Soul	Don't Stop 'Til You Get Enough	Michael Jackson	0.42	0.34	0.33	0.31
60	R&B/Soul	You Send Me	Sam Cooke	0.36	0.44	-0.11	0.56

Footnotes

1. A preliminary version of the committee machine described here was reported in Vempala & Russo (2013). Although this prior work was informed by the same theoretical framework, the computational model was based on only 12 excerpts of classical music. Given this small number of excerpts and the lack of genre diversity, the generalizability of the model was extremely limited.

2. The current study utilizes mean responses (emotion judgments and physiological responses); continuous ratings will be modeled in a separate study.
